# mSphere of Influence: the Rise of Artificial Intelligence in Infection Biology

**DOI:** 10.1128/mSphere.00315-19

**Published:** 2019-06-26

**Authors:** Artur Yakimovich

**Affiliations:** a MRC-Laboratory for Molecular Cell Biology, University College London, London, United Kingdom

**Keywords:** anthrax, artificial intelligence, bioimage analysis, computer vision, convolutional neural networks, deep learning, label-free imaging, machine learning

## Abstract

Artur Yakimovich works in the field of computational virology and applies machine learning algorithms to study host-pathogen interactions. In this mSphere of Influence article, he reflects on two papers “Holographic Deep Learning for Rapid Optical Screening of Anthrax Spores” by Jo et al. (Y. Jo, S. Park, J. Jung, J. Yoon, et al., Sci Adv 3:e1700606, 2017, https://doi.org/10.1126/sciadv.1700606) and “Bacterial Colony Counting with Convolutional Neural Networks in Digital Microbiology Imaging” by Ferrari and colleagues (A. Ferrari, S. Lombardi, and A. Signoroni, Pattern Recognition 61:629–640, 2017, https://doi.org/10.1016/j.patcog.2016.07.016). Here he discusses how these papers made an impact on him by showcasing that artificial intelligence algorithms can be equally applicable to both classical infection biology techniques and cutting-edge label-free imaging of pathogens.

## Abstract (precis)

Artur Yakimovich works in the field of computational virology and applies machine learning algorithms to study host-pathogen interactions. In this mSphere of Influence article, he reflects on two papers “Holographic Deep Learning for Rapid Optical Screening of Anthrax Spores” by Jo et al. (Y. Jo, S. Park, J. Jung, J.

## COMMENTARY

Modern day microscopy creates an incredible opportunity for infection biology. Pathogens that required complex visualization techniques just a decade ago can now be readily visualized using benchtop equipment (1–4). Notably, label-free techniques may in some cases lower, or even eliminate, the need for pathogen-specific reagents (5–7). Inevitably, the availability and abundance of pathogen and host-pathogen microscopy data now necessitate unbiased quantification and highly parallel automation to approach the depth of information contained in these images. Recent advances in machine learning (ML) and deep learning (DL) offer the possibility of novel image analysis, quantification, and classification methods (8). Dubbed artificial intelligence (AI) algorithms, according to the name of the computer science discipline, ML/DL allows for unprecedented ways of detection though pattern recognition. Two articles that highlight the power of AI for infection biology, “Holographic Deep Learning for Rapid Optical Screening of Anthrax Spores” (9) and “Bacterial Colony Counting with Convolutional Neural Networks in Digital Microbiology Imaging” (10) applied novel algorithms and label-free holographic image acquisition to bacterial spores and colonies, respectively. These two early examples showcased the flexibility of AI to tackle image analysis problems across divergent size scales.

**FIG 1:**
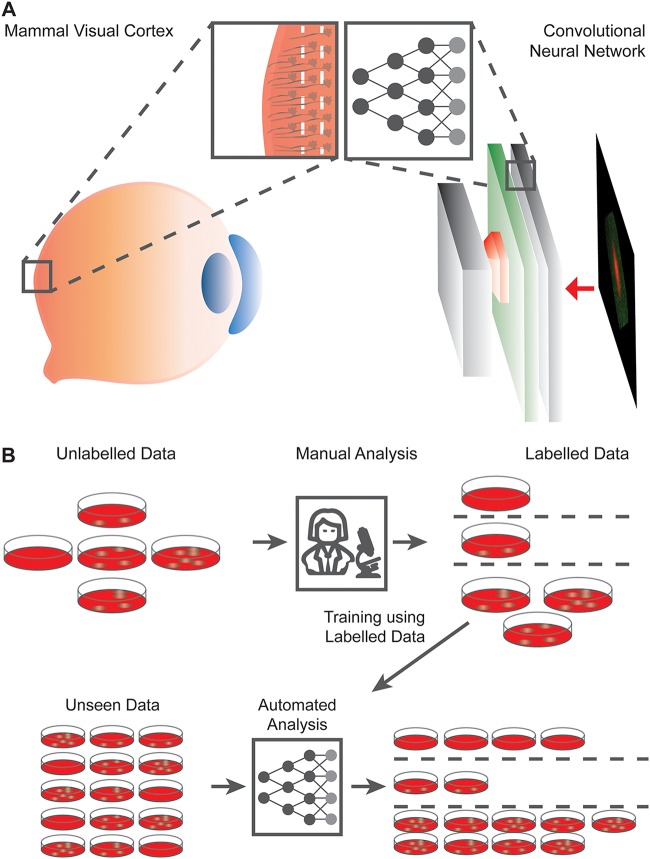
Simplistic illustration of an artificial intelligence algorithm and its applications in the infection biology data analysis. (A) Illustration of the Convolutional Neural Network (CNN) (right-hand side) analogy to mammalian visual cortex (left-hand side). Layers of biological neurons are known to provide an increasing amount of resolution, easing the processing of the visual information. Similarly, CNN relies on digital image information transformed through multiple convolution operations using layers of artificial neurons (depicted as colored boxes). Such processing is creating higher dimensional representations of a complex image. These representations can then be used in CNN training e.g., to distinguish between different class of images with higher precision. (B) Principle scheme of artificial intelligence algorithms application to infection biology problems improving analysis scalability and precision. This requires obtaining a digital input (e.g., image, sound recording etc.). Next such input is annotated by the lab’s trained specialists to obtain the desired out. Finally, an artificial neural network algorithm is devised to map the input and output with high accuracy.

For optical screening of anthrax spores, Jo and colleagues (9) demonstrate how a DL algorithm learns subtle patterns in order to accurately distinguish spores from Bacillus anthracis and four related species. Notably, images used in the study were obtained by label-free holographic microscopy, which does not rely on any biochemical or immunohistochemical labeling. While this imaging technique posed an additional challenge for the DL algorithm, it served to extend the applicability of the methodology. The trained model, called HoloConvNet, is comprised of an artificial neural network (ANN) accompanied by the list of weights and biases (i.e., parameters) obtained in the training. The HoloConvNet architecture, a so-called convolutional ANN (CNN), is inspired by the mammal visual cortex. CNNs learn a magnitude of subtle image features through their transformed representations (Fig. 1A). Using this methodology, the authors could distinguish B. anthracis from Bacillus thuringiensis, B. cereus, B. atrophaeus, and B. subtilis with greater than 96% accuracy. The holographic microscopy used in this study proved to be critical. Not only did it reveal the refractive index distribution of the various spores to be the characteristic signature on which the HoloConvoNet based its high detection precision, but by avoiding the need for specific labeling reagents, the trained DL model can now be used in various laboratories across the world.

Changing scales, Ferrari et al. (10) developed CNN-assisted bacterial colony counting as opposed to distinguishing specific pathogens. Importantly, the authors demonstrate improved detection and separation of bacterial colonies using conventional photographs of laboratory samples (10). For this, a specialist-annotated image segmentation data set was created using images of bacterial colonies from 28,500 blood agar plates. While the size of the data set used in the study illustrates the “data-hungriness” problem associated with CNNs, at 92% accuracy, the model developed is unprecedented. Remarkably, the CNN devised here is considered relatively shallow by today’s standards, suggesting that the accuracy may be further improved using the same training data set.

These papers highlight the fact that deep ANNs are great approximators for virtually any nonlinear classification problem. What most influenced me was the potential of AI to transform data analysis and drive new discovery. As the vast majority of such problems in biology are currently solved manually, a typical strategy of leveraging new AI opportunities in the lab involves replacing tedious manual tasks performed by a specialist with automated CNN workflow (Fig. 1B). For instance, the work of Jo and colleagues (9) was focused on single pathogens as data points, rather than single images, allowing for data set multiplexing. We have employed a comparable pathogen-centric view, while building our classifier for Toxoplasma gondii host-pathogen interactions (11).

A vast amount of challenges currently hinder the progress of image analysis in the field of infection biology. In this sense, infection biology represents an incredibly fertile ground for a domain-specific application of AI. Given that a high-resolution image capturing a single host cell can readily contain 10- to 1,000-fold more pathogens, it is easy to envision pathogen classification as a large data set ML problem where AI algorithms can demonstrate their edge. A multitude of recent biomedical publications following this pattern suggest the immense potential of AI to improve precision, ease the burden of repetitive manual quantification, and accelerate research discovery. Given the scale, even conservative improvements in routine tasks may well signal the dawn of a new era for infection biology, the era of machines.
